# Supplementary material for: Influence of Nitrogen-Modified Atmosphere Storage on Lipid Oxidation of Peanuts: From a Lipidomic Perspective
Source: Foods. 2024 Jan 16;13(2):277. doi: 10.3390/foods13020277 (PMC10814783; doi:10.3390/foods13020277)
Supplement: Supplementary file 1 [file foods-13-00277-s001.zip › foods-2791716-supplementary/supplementary/foods-2791716-supplementary.pdf]

Table S1. Significantly different metabolites in comparison groups of CS 300 d and 0 d based on the ANOVA test analysis and OPLS-DA analysis

Lipid Types	Subclass	Compound Name	p value	VIP
Glycerophospholipids	LPC	LPC(18:1)	0.0000	1.9095
	OXPC	OxPC(16:0/20:3+2O(1Cyc))	0.0350	1.2924
	PA	PA(18:1/18:1)	0.0136	1.2782
	PC	PC(16:0/18:2)	0.0436	1.1576
		PC(18:0/27:0)	0.0178	1.2869
	PE	PE(22:6e/7:0)	0.0030	1.6173
	PEtOH	PEtOH(13:1/20:1)	0.0355	1.3255
		PEtOH(15:0/20:4)	0.0019	1.5077
		PEtOH(16:1/19:2)	0.0044	1.5301
		PEtOH(24:0/24:0)	0.0216	1.4232
		PEtOH(26:0/26:0)	0.0005	1.7802
		PEtOH(26:0/26:1)	0.0003	1.7829
		PEtOH(27:0/22:2)	0.0277	1.3231
	PI	PI(18:0/18:1)	0.0369	1.2928
		PI(21:0/26:0)	0.0037	1.4704
		PI(27:0/20:5)	0.0035	1.6310
	BMP	BMP(26:0/26:0)	0.0003	1.7624
		BMP(4:0/18:1)	0.0140	1.4313
	HBMP	HBMP(12:0/12:0/22:0)	0.0001	1.8129
		HBMP(12:0/20:3/20:3)	0.0003	1.7459
		HBMP(14:0/14:0/20:1)	0.0320	1.1174
		HBMP(14:0/14:0/22:1)	0.0323	1.1212
		HBMP(14:0/18:0/18:0)	0.0054	1.3995
		HBMP(14:1/18:0/18:0)	0.0016	1.6940
		HBMP(14:1/18:1/18:1)	0.0040	1.5297
		HBMP(16:0/16:0/16:2)	0.0000	1.8964
		HBMP(16:0/16:0/20:3)	0.0095	1.4202
	DGTS	DGTS(16:3/24:4)	0.0000	1.8724
		DGTS(18:1/26:4)	0.0079	1.3275
		DGTS(20:2/26:4)	0.0001	1.7253
		DGTS(24:0/27:0)	0.0159	1.4930
		DGTS(24:2/26:4)	0.0026	1.6183
		DGTS(25:0/22:4)	0.0000	1.8737
		DGTS(25:0/24:2)	0.0120	1.4501
		DGTS(26:1/26:1)	0.0080	1.5213
		DGTS(26:2/22:5)	0.0138	1.4632
		DGTS(26:2/26:4)	0.0101	1.4764
		DGTS(27:0/16:2)	0.0313	1.3777
		DGTS(27:0/18:3)	0.0004	1.7755
		DGTS(27:0/18:4)	0.0003	1.7422

		DGTS(27:0/20:5)	0.0013	1.5642
		DGTS(27:0/22:3)	0.0008	1.6398
		DGTS(27:0/22:4)	0.0017	1.5721
		DGTS(27:0/24:1)	0.0007	1.7644
		DGTS(27:0/24:2)	0.0080	1.4864
		DGTS(27:0/26:1)	0.0279	1.2956
		DGTS(27:0/26:2)	0.0102	1.5257
		DGTS(8:0/22:6)	0.0003	1.6620
	DAG	DAG(16:0/18:2)	0.0188	1.3869
		DAG(18:0/18:1)	0.0000	1.8875
	SQDG	SQDG(20:0/26:1)	0.0013	1.6513
		SQDG(23:0/23:0)	0.0009	1.6939
		SQDG(27:0/15:1)	0.0089	1.4291
Glycerolipids	TAG	TAG(12:0/12:0/12:1)	0.0000	1.8986
		TAG(12:0/12:0/12:2)	0.0000	1.8015
		TAG(12:0/16:3/18:0)	0.0021	1.5204
		TAG(12:0/16:3/22:0)	0.0057	1.5403
		TAG(12:0/21:2/21:2)	0.0041	1.5550
		TAG(12:0/22:2/22:2)	0.0005	1.6725
		TAG(12:1/13:1/18:1)	0.0141	1.4104
		TAG(12:1/22:1/22:1)	0.0012	1.5973
		TAG(12:3/12:3/13:0)	0.0233	1.2201
		TAG(12:3/22:1/22:1)	0.0004	1.7363
		TAG(13:1/22:3/22:3)	0.0460	1.0631
		TAG(14:0/16:0/18:1)	0.0342	1.1880
		TAG(14:0/16:0/18:2)	0.0123	1.4265
		TAG(14:0/18:0/18:0)	0.0021	1.5423
		TAG(14:0/18:1/18:2)	0.0120	1.4227
		TAG(14:0/18:2/18:2)	0.0346	1.2365
		TAG(14:0/20:1/20:1)	0.0041	1.4754
		TAG(14:0/20:5/22:1)	0.0357	1.1742
		TAG(14:0/22:1/22:1)	0.0001	1.7810
		TAG(14:1/20:0/22:4)	0.0002	1.7518
		TAG(14:1/22:2/22:2)	0.0007	1.7077
		TAG(14:3/20:0/20:0)	0.0180	1.4008
		TAG(14:3/22:3/22:4)	0.0003	1.7498
		TAG(15:0/15:0/22:3)	0.0000	1.8717
		TAG(15:0/21:5/22:3)	0.0018	1.6166
		TAG(15:1/15:1/22:1)	0.0000	1.9278
		TAG(15:1/21:4/21:5)	0.0468	1.1203
		TAG(15:1/22:4/22:4)	0.0145	1.4459
		TAG(15:2/19:0/21:5)	0.0000	1.7963
		TAG(15:2/22:3/22:3)	0.0243	1.3224
		TAG(15:2/22:3/22:4)	0.0179	1.2432

	TAG(15:3/15:3/19:2)	0.0011	1.6971
	TAG(15:3/19:0/21:5)	0.0018	1.5990
	TAG(15:3/21:4/21:4)	0.0073	1.5060
	TAG(15:3/22:0/22:6)	0.0470	1.1547
	TAG(15:3/22:1/22:7)	0.0368	1.3049
	TAG(15:3/22:3/22:3)	0.0010	1.6963
	TAG(15:3/22:4/22:4)	0.0380	1.3230
	TAG(16:0/16:0/18:1)	0.0000	1.9023
	TAG(16:0/16:0/18:2)	0.0000	1.9265
	TAG(16:0/16:0/20:1)	0.0080	1.4900
	TAG(16:0/16:0/22:1)	0.0071	1.4049
	TAG(16:0/16:0/22:2)	0.0352	1.3127
	TAG(16:0/17:3/20:6)	0.0109	1.5202
	TAG(16:0/18:1/18:3)	0.0049	1.4845
	TAG(16:0/19:1/19:1)	0.0243	1.2954
	TAG(16:0/22:2/22:2)	0.0011	1.6464
	TAG(16:1/18:2/18:2)	0.0000	1.9601
	TAG(16:2/18:1/18:1)	0.0038	1.6048
	TAG(16:2/18:2/18:2)	0.0062	1.5700
	TAG(16:3/18:1/18:1)	0.0015	1.6886
	TAG(16:3/20:0/22:5)	0.0121	1.3462
	TAG(16:4/18:0/21:5)	0.0003	1.7188
	TAG(16:4/20:1/20:1)	0.0199	1.3961
	TAG(16:4/21:0/22:7)	0.0056	1.5159
	TAG(16:5/19:0/19:0)	0.0001	1.8058
	TAG(17:0/22:3/22:3)	0.0185	1.3275
	TAG(17:1/17:1/21:5)	0.0002	1.7347
	TAG(17:1/17:1/22:4)	0.0075	1.4036
	TAG(17:1/19:0/20:6)	0.0004	1.7056
	TAG(17:1/22:1/22:1)	0.0014	1.5844
	TAG(17:2/17:2/20:0)	0.0001	1.7462
	TAG(17:2/18:5/20:0)	0.0042	1.4753
	TAG(17:2/19:1/22:7)	0.0441	1.2971
	TAG(17:2/20:0/20:6)	0.0014	1.5831
	TAG(17:2/20:0/22:6)	0.0001	1.7896
	TAG(17:2/22:7/22:7)	0.0016	1.6408
	TAG(17:3/18:5/20:0)	0.0164	1.3253
	TAG(17:3/19:3/19:3)	0.0098	1.4401
	TAG(17:3/19:3/20:2)	0.0482	1.2329
	TAG(17:3/20:0/20:6)	0.0004	1.7121
	TAG(17:3/21:0/22:7)	0.0458	1.2940
	TAG(17:3/22:6/22:6)	0.0250	1.3518
	TAG(18:0/19:0/20:6)	0.0006	1.6339
	TAG(18:0/22:2/22:2)	0.0127	1.3338

		TAG(18:1/18:1/22:7)	0.0166	1.4327
		TAG(18:1/18:4/21:5)	0.0219	1.4172
		TAG(18:1/18:5/19:3)	0.0217	1.3103
		TAG(18:2/19:0/21:5)	0.0492	1.1320
		TAG(18:2/21:5/22:0)	0.0002	1.7813
		TAG(18:3/18:3/19:2)	0.0017	1.6234
		TAG(18:3/19:0/20:6)	0.0000	1.8070
		TAG(18:3/19:4/20:0)	0.0096	1.4917
		TAG(18:3/20:0/22:5)	0.0086	1.4375
		TAG(18:4/18:4/19:0)	0.0292	1.2406
		TAG(18:4/18:4/19:1)	0.0001	1.8513
		TAG(18:4/18:4/21:0)	0.0278	1.3758
		TAG(18:4/19:0/20:6)	0.0014	1.6094
		TAG(18:4/19:0/22:6)	0.0002	1.7671
		TAG(18:4/19:2/21:4)	0.0002	1.7316
		TAG(18:5/19:0/20:6)	0.0062	1.3852
		TAG(18:5/20:0/21:2)	0.0001	1.7826
		TAG(18:5/22:0/22:5)	0.0016	1.5991
		TAG(19:0/19:3/20:6)	0.0000	1.8423
		TAG(19:0/20:3/20:6)	0.0003	1.7498
		TAG(19:0/21:4/21:4)	0.0004	1.6569
		TAG(19:0/22:2/22:6)	0.0002	1.7928
		TAG(19:1/19:1/22:6)	0.0299	1.2536
		TAG(19:1/20:1/22:7)	0.0011	1.6448
		TAG(19:1/21:5/21:5)	0.0047	1.5956
		TAG(19:2/19:2/22:5)	0.0475	1.1654
		TAG(19:2/20:3/20:6)	0.0452	1.2902
		TAG(19:2/22:0/22:7)	0.0000	1.8325
		TAG(19:3/20:0/20:5)	0.0000	1.8342
		TAG(19:5/19:5/20:0)	0.0164	1.3824
		TAG(20:0/20:4/21:5)	0.0004	1.7123
		TAG(20:0/20:6/21:2)	0.0003	1.7255
		TAG(20:0/22:2/22:5)	0.0000	1.8685
		TAG(20:1/21:5/22:3)	0.0001	1.8160
		TAG(20:3/20:3/20:3)	0.0089	1.4520
		TAG(20:3/21:4/22:1)	0.0002	1.7418
		TAG(20:5/20:5/21:4)	0.0055	1.5277
		TAG(21:0/21:1/22:7)	0.0009	1.6819
Fatty acyls	ACar	ACar(24:2)	0.0000	1.8776
	FA	FA(18:1)	0.0140	1.3921
	FAHFA	FAHFA(2:0/16:1)	0.0154	1.4275
		FAHFA(9:0/10:0)	0.0033	1.4245
Sphingolipids	Cer	Cer/ADS(d23:0/19:1)	0.0341	1.3102
		Cer/AP(t18:0/24:0)	0.0489	1.1544

		Cer/AP(t18:1/22:0)	0.0371	1.2107
		HexCer/NS(d14:2/24:2)	0.0096	1.3859
		HexCer/NS(d14:2/26:2)	0.0033	1.5438
		HexCer/NS(d14:3/24:2)	0.0012	1.6260
		HexCer/NS(d14:3/26:2)	0.0103	1.4608
	HexCer	HexCer/NS(d14:3/36:2)	0.0008	1.6545
		HexCer/NS(d14:3/42:1)	0.0004	1.6979
		HexCer/NS(d17:1/16:2)	0.0030	1.6070
		HexCer/NS(d25:2/34:2)	0.0008	1.6432
		HexCer/NS(d29:2/30:2)	0.0003	1.6961
		GlcADG(12:0/17:1)	0.0192	1.3283
	GlcADG	GlcADG(14:0/14:0)	0.0272	1.2478
Glycolipids		GlcADG(24:2/24:2)	0.0253	1.2530
	AcylGlcADG	AcylGlcADG(18:0/18:0/18:2)	0.0009	1.6786

Table S2. Significantly different metabolites in comparison groups of NS 300 d and CS 300 d based on the ANOVA test analysis and OPLS-DA analysis

Lipid Types	Subclass	Compound Name	p value	VIP
Glycerophospholipids	LPC	LPC(18:1)	0.0015	2.7160
	OxPE	OxPE(18:0/18:1+1O(1Cyc))	0.0482	1.9762
		OxPE(18:1/18:0+1O(1Cyc))	0.0426	1.9876
	PEtOH	PEtOH(27:0/19:2)	0.0418	2.0783
	PA	PA(16:0/18:1)	0.0273	2.1763
	DGTS	DGTS(24:4/26:4)	0.0286	2.1563
		DGTS(27:0/22:4)	0.0024	2.6751
		DGTS(3:0/26:1)	0.0458	2.0002
	HBMP	HBMP(14:0/14:0/20:1)	0.0248	2.1326
		HBMP(14:0/14:0/22:1)	0.0049	2.5667
Glycerolipids	DAG	DAG(18:1/18:3)	0.0474	2.0025
	SQDG	SQDG(20:0/26:2)	0.0382	2.0460
	TAG	TAG(14:0/21:0/22:7)	0.0031	2.6315
		TAG(15:0/21:5/22:3)	0.0105	2.4200
		TAG(16:3/21:0/22:7)	0.0469	1.9771
		TAG(18:0/18:0/18:1)	0.0487	1.9726
		TAG(18:1/18:1/18:2)	0.0237	2.1732
		TAG(18:4/19:0/20:6)	0.0299	2.0613
		TAG(19:0/22:2/22:6)	0.0436	1.9985
		TAG(19:1/20:2/20:6)	0.0491	2.0062
		TAG(20:0/20:6/21:2)	0.0058	2.5333
		TAG(20:1/21:5/22:3)	0.0460	1.9767
Sphingolipids	Cer	Cer/AP(t18:1/26:0)	0.0270	2.1145
Fatty acyls	FA	FA(20:0)	0.0017	2.7489
		FA(20:1)	0.0126	2.4106
	FAHFA	FAHFA(9:0/10:0)	0.0014	2.7501
glycolipids	GlcADG	GlcADG(22:0/23:0)	0.0140	2.3035
		GlcADG(24:2/24:2)	0.0209	2.2017
